# Glucose uptake in pigment glia suppresses Tau-induced inflammation and photoreceptor degeneration

**DOI:** 10.1242/dmm.052057

**Published:** 2025-04-29

**Authors:** Mikiko Oka, Sho Nakajima, Emiko Suzuki, Shinya Yamamoto, Kanae Ando

**Affiliations:** ^1^ Department of Biological Sciences, Tokyo Metropolitan University, Tokyo 192-0397, Japan; ^2^ Department of Molecular and Human Genetics, Baylor College of Medicine, Houston, TX 77030, USA; ^3^ Jan and Dan Duncan Neurological Research Institute for Texas Children's Hospital, Houston, TX 77030, USA

**Keywords:** *Drosophila*, Neurodegeneration, Glia, Inflammation, Glucose

## Abstract

Brain inflammation contributes to the pathogenesis of neurodegenerative diseases, such as Alzheimer's disease (AD). Glucose hypometabolism and glial activation are pathological features seen in AD brains; however, the connection between the two is not fully understood. Using a *Drosophila* model of AD, we identified that glucose metabolism in glia plays a critical role in neuroinflammation under disease conditions. Expression of human MATP (hereafter referred to as Tau) in the retinal cells, including photoreceptor neurons and pigment glia, causes photoreceptor degeneration accompanied by the formation of dark-stained round inclusion-like structures and swelling of the lamina cortex. We found that inclusion-like structures are formed by glial phagocytosis, and swelling of the laminal cortex correlates with the expression of antimicrobial peptides. Coexpression of human glucose transporter 3 (*SLC2A3*, hereafter referred to as *GLUT3*) with Tau in the retina does not affect Tau levels but suppresses these inflammatory responses and photoreceptor degeneration. We also found that expression of *GLUT3*, specifically in the pigment glia, is sufficient to suppress inflammatory phenotypes and mitigate photoreceptor degeneration in the Tau-expressing retina. Our results suggest that glial glucose metabolism contributes to inflammatory responses and neurodegeneration in tauopathy.

## Teaser

**Summary:** Glucose uptake into pigment glia cells suppresses inflammatory responses and photoreceptor degeneration in the *Drosophila* model of tauopathy.

## INTRODUCTION

Alzheimer's disease (AD) is a progressive neurodegenerative disorder and the most common cause of dementia among older people (Knopman et al., 2021). AD is characterized by the extracellular deposition of β-amyloid and intracellular accumulation of abnormally phosphorylated forms of the microtubule-associated protein Tau (MATP in humans, hereafter referred to as Tau) (Samudra et al., 2023). Neuroinflammation is another core pathology in AD and other neurodegenerative disorders (Sobue et al., 2023). Inflammatory responses in the central nervous system are mediated by mainly glial cells, including microglia and astrocytes, to protect against infections and injuries; however, under neurodegenerative disease conditions, glial cells lose their neuroprotective functions and acquire reactive phenotypes (Ransohoff, 2016; Streit, 2002). Although they can remove protein aggregates by phagocytosis, activated glial cells can also harm neurons via excess phagocytosis and the release of neurotoxic cytokines (Chen and Holtzman, 2022). However, what triggers glial activation under disease conditions is not fully understood.

A decline in brain glucose metabolism has been pathologically linked to AD (Butterfield and Halliwell, 2019). Most of the ATP required to support brain function is supplied by glucose metabolism (Bélanger et al., 2011), and glucose utilization is reduced in the brains of AD patients (Dukart et al., 2013; Huang et al., 2020; Oka et al., 2021). Dysregulated glucose metabolism in AD may be due to cerebral hypoperfusion (Park et al., 2019) or downregulation of glucose transporter proteins (GLUTs) that mediate glucose uptake across the blood-brain barrier and delivery to glial cells (Bélanger et al., 2011; Koepsell, 2020; Kyrtata et al., 2021; Liu et al., 2008). Glial cells also rely on glucose, and cellular metabolism can regulate their activation (Wang et al., 2019; Xiang et al., 2021). The mitochondrial oxidative phosphorylation is reduced in the activated microglial cells (Nair et al., 2019), and mitochondrial dysfunction in astrocytes has been reported in amyotrophic lateral sclerosis and neuroinflammation models (Fiebig et al., 2019; Martínez-Palma et al., 2019). While these reports suggest that targeting glial metabolism is a strategy to decrease neuroinflammation and suppress neurodegeneration, mechanistic investigation in an *in vivo* model is required to explore this possibility.

Here, we investigated the roles of glucose metabolism in glial cells on Tau-induced neurodegeneration in a *Drosophila* model. We identified glia-associated phenotypes in the fly retina expressing human Tau, such as inclusion-like structures by phagocytosis and induction of antimicrobial peptide (AMP) expression. *Drosophila* retina contains neurons and several glial cells, and we found that pigment glia plays a critical role in Tau-induced photoreceptor degeneration. Enhancement of glucose uptake in pigment glial cells ameliorates Tau-induced laminal cortex swelling as well as photoreceptor degeneration. Our results suggest that glucose hypometabolism in glial cells contributes to neurodegeneration in tauopathy.

## RESULTS

### Tau expression in the fly retina causes photoreceptor degeneration, swelling of the laminal cortex and inclusion formation

**Fig. 1.**
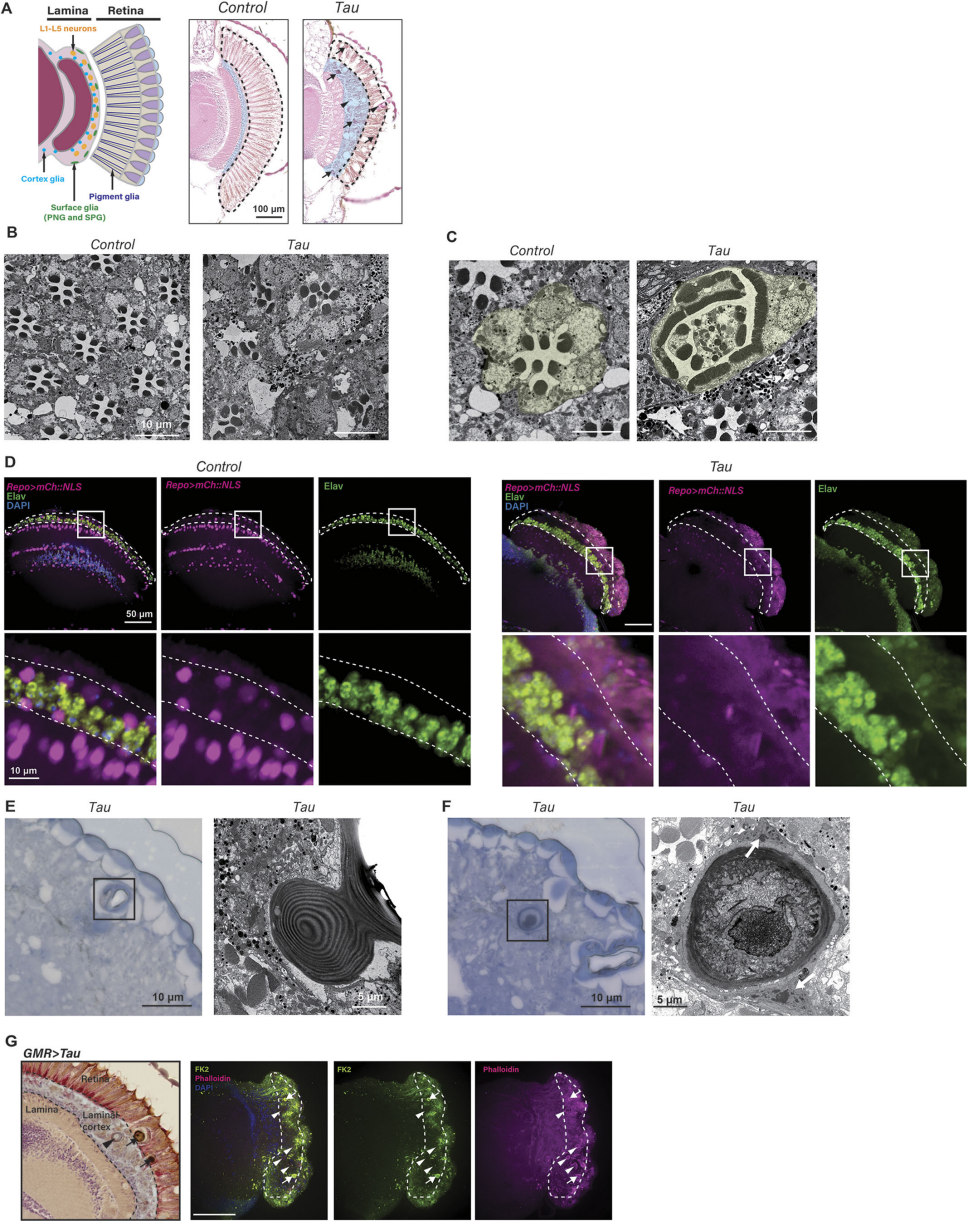
**Expression of human Tau in fly retina causes photoreceptor degeneration, swelling of the laminal cortex and the formation of inclusions.** (A) Schematic representation of the retina (left) and H&E-stained retinal sections (right). Expression of human Tau causes degeneration, which is indicated by vacuoles in the retina (area encircled by the dashed line), swelling of the laminal cortex (blue) and inclusions (arrows and arrowheads). Tau expression was driven by *GMR-GAL4* and luciferase was used as a control (Control). Flies were 5 days old. Scale bar: 100 µm. (B-C) Representative transmission electron microscopy (TEM) images of eyes obtained from 5-day-old flies expressing luciferase (*Control*) or human Tau (*Tau*). Tau expression showed photoreceptor degeneration with distorted rhabdomeres (yellow shade). (D) Tau expression increases neurons and reduces glial cells in the laminal cortex. Glial cells were labeled with Repo>mCh::NLS (magenta), neurons were immunolabeled with an anti-elav antibody (green). DAPI was used to stain nuclei (blue). To analyze the effect of tau expression in the retinal cells, *GMR-Tau (P301L)* that expresses human Tau with the pathological P301L mutation directly under the control of the GMR regulatory sequence, was combined with Repo>mCh::NLS (Tau). The laminal cortex is outlined by dotted lines. Boxed areas within the top images are shown magnified underneath. Scale bar: 50 µm top images) and 10 µm (bottom images). (E,F) Toluidine Blue staining and TEM retinal images obtained from flies as described in A, showing inclusions in the Tau-expressing retina. Tau expression showed two types of inclusion, i.e. invaginated corneal lens (E, arrowheads in A) and debris-containing inclusions wrapped by glial cells (F, arrows in A). (G) Left: a paraffin-embedded section of a fly retina expressing human Tau. The arrowhead points to the invaginated corneal lens, while the arrows indicate electron-dense inclusions. Right: Images of a Tau-expressing retina immunostained with anti-ubiquitin antibody (FK2, green). Filamentous actin was stained with phalloidin (magenta), nuclei were stained with DAPI (blue). Areas surrounded by the dotted line indicate the laminal cortex. Scale bar: 50 µm.

Expression of human Tau in *Drosophila* by using the pan-retinal eye-specific *GMR-GAL4* driver (Freeman, 1996) causes a rough-eye phenotype in the compound eye surface accompanied by the formation of vacuoles caused by apoptosis in the retina and axonal degeneration in the lamina (Fig. 1A) (Iijima-Ando et al., 2012; Jackson et al., 2002; Wittmann et al., 2001). UAS-Tau transgene alone does not cause these phenotypes, as described previously (Wittmann et al., 2001 and Fig. S1). Transmission electron microscopy (TEM) revealed degenerating photoreceptor neurons with disoriented rhabdomeres in Tau-expressing flies (Fig. 1B,C, ommatidium colored in yellow). We also noticed swelling of the laminal cortex (shaded in blue in Fig. 1A) that is located between the lamina and retina, and consists of layers of L1-L5 neurons, surface glia – i.e. perineurial glia (PNG) and subperineurial glia (SPG) − and cortex glia (Chotard and Salecker, 2007; Kremer et al., 2017). We analyzed neuronal and glial cell distribution in the laminal cortex by using glial expression of mCherry carrying the nuclear localization signal (mCh::NLS) and immunostaining for the neuronal marker protein elav (Fig. 1D). In the laminal cortex, surface glia and cortex glia form two layers, and cell bodies of L1-L5 neurons another layer. With Tau expression in the retina, the neuronal layers in the laminal cortex were enlarged by an increased number of neurons. In contrast, glial cells in the laminal cortex were degenerated (Fig. 1D).

Tau expression caused the formation of dense inclusion-like structures in the retina and lamina (Fig. 1A). TEM analysis of these structures revealed two distinct ultrastructures: an invaginated corneal lens (Fig. 1A,E,G, arrowheads) as well as dark inclusions containing cellular debris (Fig. 1A,F,G, arrows) wrapped by glia (Fig. 1F, white arrows). These inclusions contain ubiquitylated proteins and cytoskeleton proteins, such as filamentous actin (Fig. 1G), suggesting that they are dead cells that harbor abnormal protein accumulation and are engulfed by glia. These results suggest that glial cells are also affected by Tau protein expression.

### Tau expression induces glial phagocytosis and expression of AMPs

**Fig. 2.**
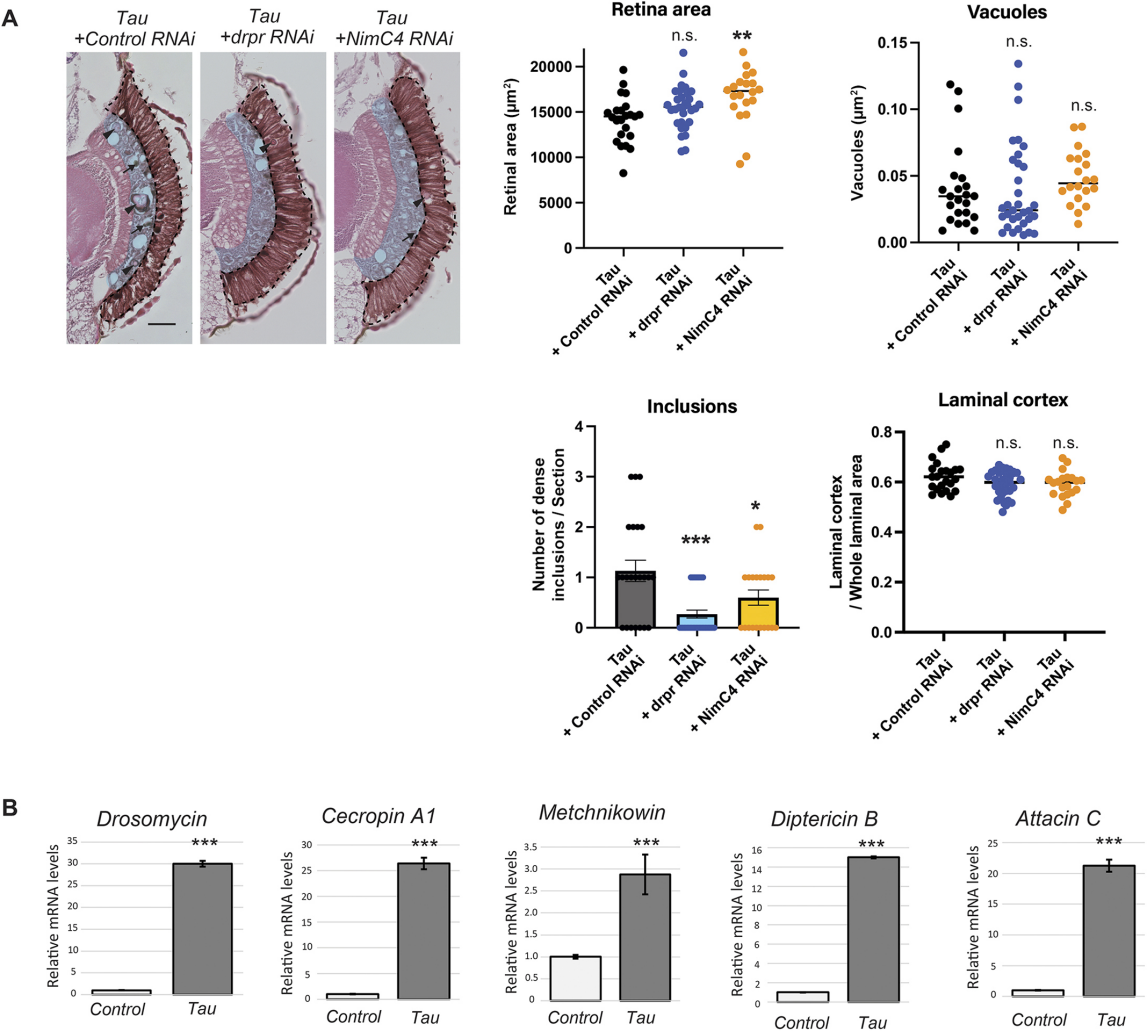
**Tau expression induces glial phagocytosis and expression of AMPs.** (A) Inclusions in the Tau-expressing retina are formed by glial phagocytosis. RNAi-mediated knockdown of *drpr* or *NimC4* reduced the number of inclusions in the Tau-expressing eyes. Representative images of H&E-stained tissue sections and quantification of the retinal area, vacuoles, inclusions and laminal cortex as indicated. Scale bar: 100 µm. Data are plotted as the mean±s.e., *n*=20-34, ***P<0.001, **P<0.01, *P<0.05; n.s., not significant (*P*>0.05); one-way ANOVA followed by Tukey's HSD multiple comparisons test. Flies were used at 7 days after eclosion. (B) Tau induces expression of the antimicrobial peptides (AMPs) *Drosomycin*, *Cecropin A1*, *Metchnikowin, Diptericin B* and *Attacin C*. Heads of flies either expressing *GMR-GAL4* driven luciferase (Control) or human Tau (Tau) were subjected to qRT-PCR to quantify expression of the indicated AMPs. Mean±s.e., *n*=3, ***P<0.001. Statistical significance was assessed using unpaired two-tailed *t*-test. Flies were used at 7 days after eclosion.

The appearance of inclusion-like structures (Fig. 1F) was similar to that of glial bodies, membranous structures that represent abnormal multilayered wrappings by glial cells (Dutta et al., 2016). We were motivated to test if these inclusions are formed through glial phagocytosis. Glial phagocytic activities in the adult fly brain are mediated by the two glial transmembrane phagocytic receptors Draper (Drpr) and Nimrod C4 (NimC4, also known as SIMU) (Kurant et al., 2008; Scheib et al., 2012). We found that blocking phagocytosis in the retina by RNAi-mediated knockdown of *drpr* and *NimC4* did not significantly affect the eye size or photoreceptor degeneration but reduced the number of inclusions in the tau-expressing retina (Fig. S2, Fig. 2A). These results indicate that these inclusions are formed by glial engulfment activity.

Proinflammatory responses in *Drosophila* involve the expression of AMPs downstream of Toll and immune-deficiency (Imd) pathways (Harnish et al., 2021; Hedengren et al., 1999; Sakakibara et al., 2023; Shukla et al., 2019). We found significantly elevated expression levels of AMPs, including *Drosomycin* (*Drs*), *Cecropin A1* (*CecA1*), *Metchnikowin* (*Mtk*), *Diptericin B* (*DptB*), and *Attacin C* (*AttC*), in flies that express Tau driven via *GMR-GAL4* (Fig. 2B). These results suggest that Tau expression in the fly retina induces glial phagocytosis and inflammatory responses, which are both hallmarks of glial activation.

### Enhancement of glucose uptake suppresses Tau-induced photoreceptor degeneration and swelling of the laminal cortex

**Fig. 3.**
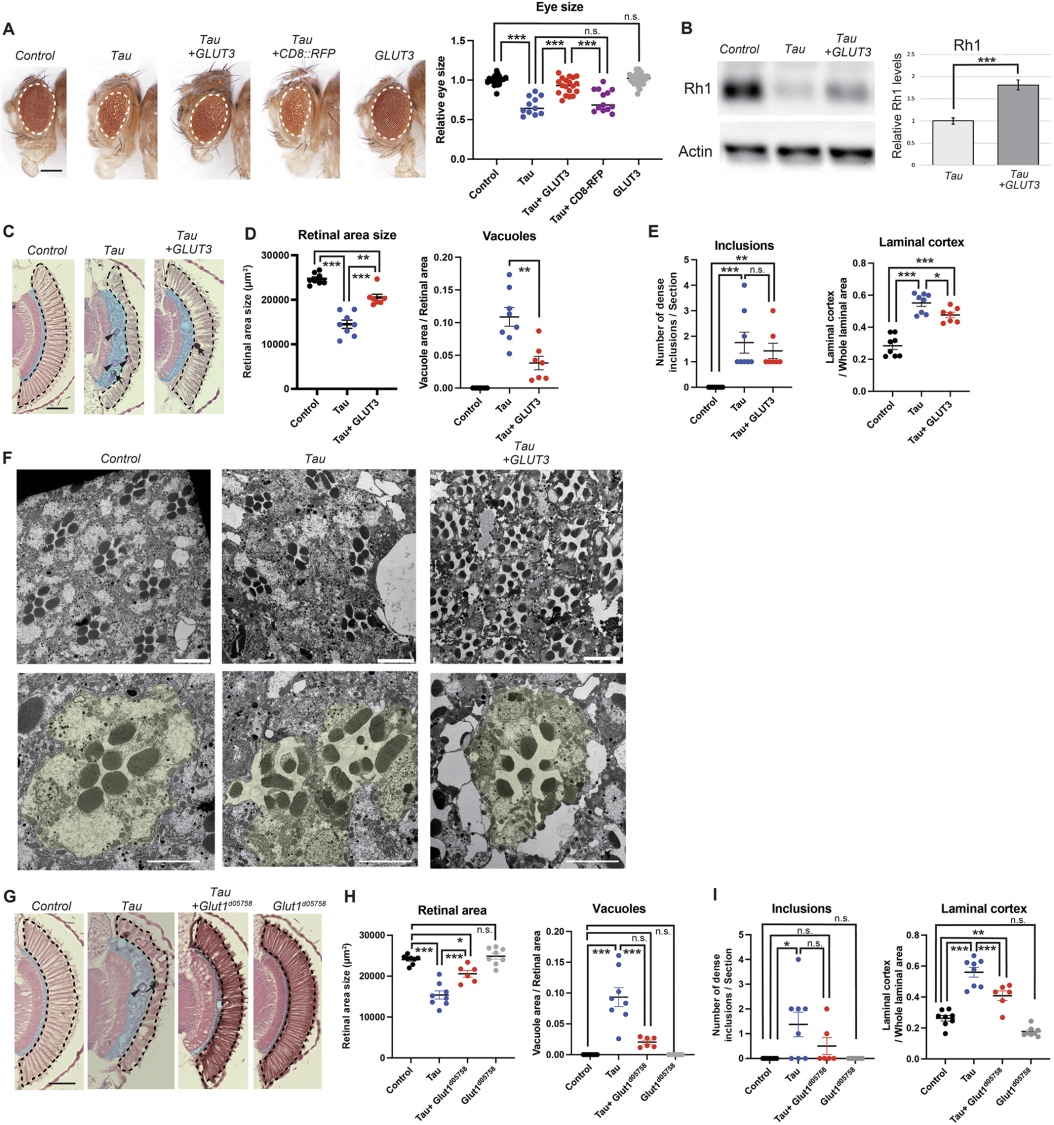
**Enhancement of glucose uptake suppresses Tau-induced photoreceptor degeneration and swelling of the laminal cortex.** (A) The reduction in the eye size caused by Tau expression (compare Control and *Tau*) was suppressed by GLUT3 expression (compare *Tau* and *Tau+GLUT3*). Expression of a control protein mCD8::ChRFP did not affect Tau-induced eye phenotype (compare *Tau* and *Tau+mCD8::ChRFP*), and expression of GLUT3 alone did not affect eye size (compare *Control and GLUT3*). Scale bar: 250 µm. Data are plotted as the mean±s.e., *n*=11-27, ***P<0.001; n.s., not significant (*P*>0.05), one-way ANOVA followed by Tukey's HSD multiple comparisons test. (B-F) Human GLUT3 expression suppresses Tau-induced photoreceptor degeneration and glial phenotype. (B) GLUT3 expression suppressed Tau-induced reduction in Rhodopsin 1 protein levels (Rh1). Fly heads expressing Tau with a control protein mCD8::ChRFP (*Tau*) or Tau and GLUT3 (*Tau+GLUT3*) were subjected to western blotting with an anti-Rh1 antibody. Actin was used as a loading control. Representative blot and quantification are shown. Mean±s.e., *n*=3, n.s., ***P<0.001. Statistical significance was assessed with unpaired two-tailed *t*-test. (C-F) GLUT3 coexpression suppressed Tau-induced neurodegeneration and glial phenotypes. (C) Representative images of sections. (D) GLUT3 coexpression suppressed Tau-induced retinal degeneration (quantification of areas of the retina vacuole). Statistical significance was assessed with unpaired two-tailed *t*-test (mean±s.e., *n*=7-9, **P<0.01, ***P<0.001). (E) GLUT3 coexpression did not affect inclusions but suppressed swelling of the laminal cortex. Statistical significance was assessed with unpaired two-tailed *t*-test (mean±s.e., *n*=7-8, *P<0.05, n.s., not significant (*P*>0.05). (F) Representative TEM images of eye photoreceptors in flies as indicated. GLUT3 coexpression mitigated rhabdomere distortion (yellow) in the eyes of flies expressing Tau. (G-I) Expression of *Drosophila Glut1* also suppressed Tau-induced retinal degeneration and swelling of the laminal cortex. Representative images of retinal sections (G) and quantification of areas of retina and vacuoles (H), as well as number of inclusions and size of the laminal cortex (I). Mean±s.e., *n*=6-8, *P<0.05, **P<0.01, ***P<0.001, one-way ANOVA followed by Tukey's HSD multiple comparisons test (G and I), n.s. (*P*>0.05). Flies were used at 7 days after eclosion. Scale bar: 100 µm.

We analyzed the effects of enhancement of glucose uptake in Tau-induced phenotypes in the retina by expressing human SLC2A3 (officially known and hereafter referred to as GLUT3), which has been shown to enhance glucose uptake in cells effectively (Besson et al., 2015; Manzo et al., 2019; Oka et al., 2021). Coexpression of GLUT3 increased the eye size in Tau flies (Fig. 3A, compare *Tau* and *Tau+GLUT3*, *P*<0.001). The eyes of flies coexpressing Tau and mCD8::ChRFP were similar to those in flies expressing Tau alone, indicating that the increase in eye size caused by coexpression of *GLUT3* is neither due to non-specific effects of an exogenous protein nor to reduction in GAL4 activity (Fig. 3A, compare *Tau* and *Tau+mCD8::ChRFP*, *P*>0.05). Expression of GLUT3 alone did not affect eye size (Fig. 3A, compare Control and *GLUT3*, *P*>0.05). Rhodopsin 1 (Rh1) protein levels are significantly lower in Tau flies, probably because the loss of photoreceptor neurons (Fig. 3B, compare Control and *Tau*). Coexpression of GLUT3 restored Rh1 protein levels (Fig. 3B, compare *Tau* and *Tau+GLUT3*, *P*<0.001). There were also significantly fewer vacuoles in the retina of flies coexpressing Tau and GLUT3 compared with that in flies expressing Tau alone (Fig. 3C-D, *P*<0.01). However, at ultrastructural levels, while the retina of Tau-expressing flies contained degenerating photoreceptor neurons with distorted rhabdomeres (Fig. 1C, ommatidium colored in yellow), ommatida of flies that coexpress Tau and GLUT3 retained rhabdomeres with proper orientation (Fig. 3F, *Tau+GLUT3*). Coexpression of GLUT3 did not affect the number of inclusion-like structures (Fig. 3E), but suppressed swelling of the laminal cortex (Fig. 3E). Overexpression of *Drosophila* Glut1 (Fig. S3), a fly homolog of human GLUT3, also suppressed tau-induced retinal degeneration and swelling of the laminal cortex (Fig. 3G-I). These results indicate that enhancement of glucose uptake suppresses Tau-induced neurodegeneration and glial phenotypes.

### Enhancement of glucose uptake does not affect Tau phosphorylation and accumulation

**Fig. 4.**
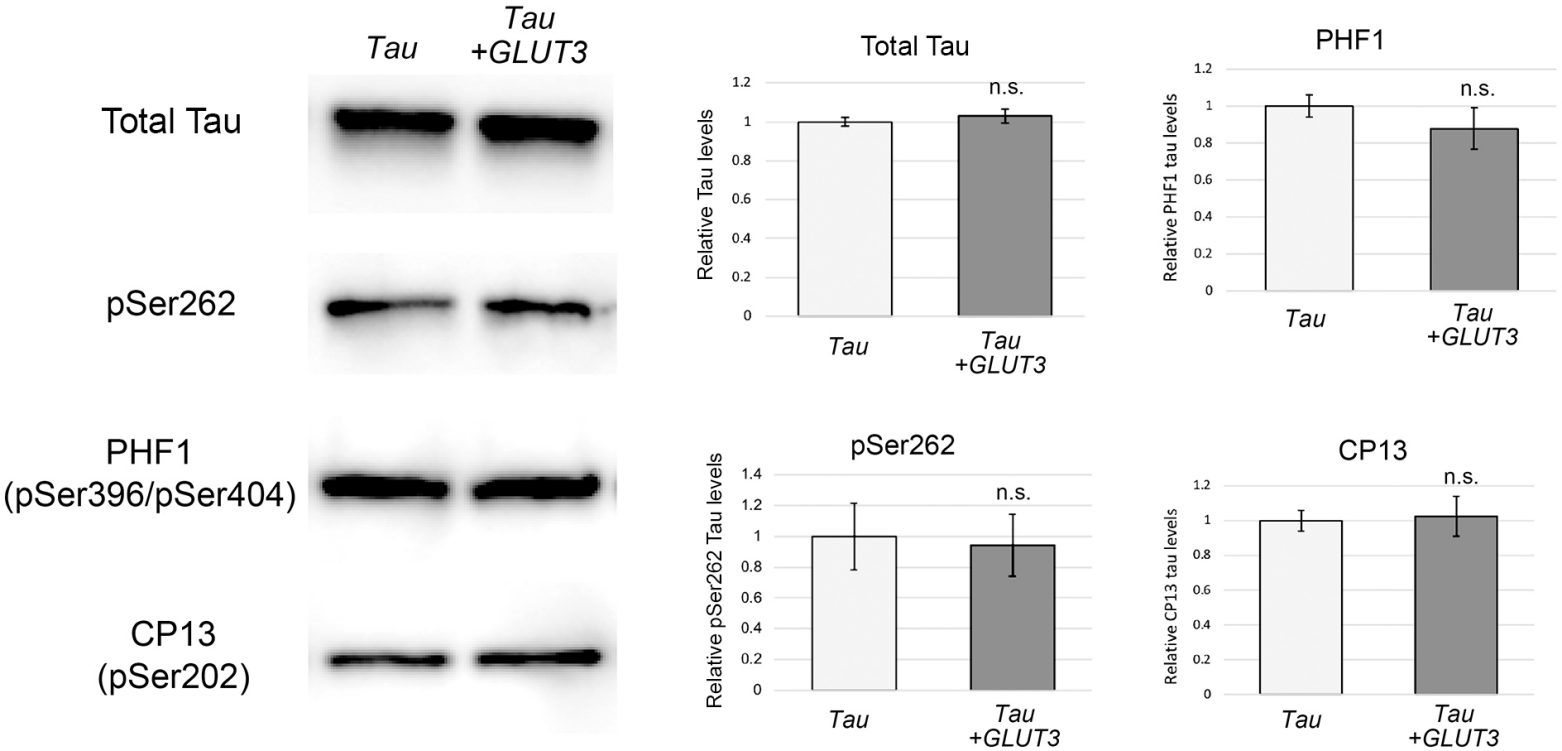
**Enhanced glucose uptake does not affect Tau phosphorylation and accumulation.** Heads obtained from flies expressing human Tau with *CD8::GFP* as a control (Tau) or those coexpressing Tau and *GLUT3* (*Tau+GLUT3*) were subjected to western blotting with anti-Tau (total Tau) or anti-phospho-Tau monoclonal antibodies (pS262, PHF1 and CP13). Actin was used as a loading control. Data are plotted as the mean±s.e., *n*=4-6, n.s., not significant (*P*>0.05). Statistical significance was assessed with unpaired two-tailed *t*-test. Flies were used at 3 days after eclosion.

To understand the mechanisms underlying the neuroprotective effects of enhanced glucose uptake, we first analyzed the effect of GLUT3 expression on phosphorylation and accumulation of Tau by using western blotting with monoclonal antibodies. Coexpression of GLUT3 did not affect the levels of total Tau (Total Tau) or its phosphorylation at disease-associated sites, including phosphorylation at Ser262 (pSer262), Ser396/404 (PHF1) or Ser202 (CP13) (Fig. 4). These results suggest that enhanced glucose uptake mitigates retinal degeneration downstream of Tau phosphorylation and accumulation.

### Enhancement of glucose uptake in pigment glial cells suppresses Tau-induced neurodegeneration

**Fig. 5.**
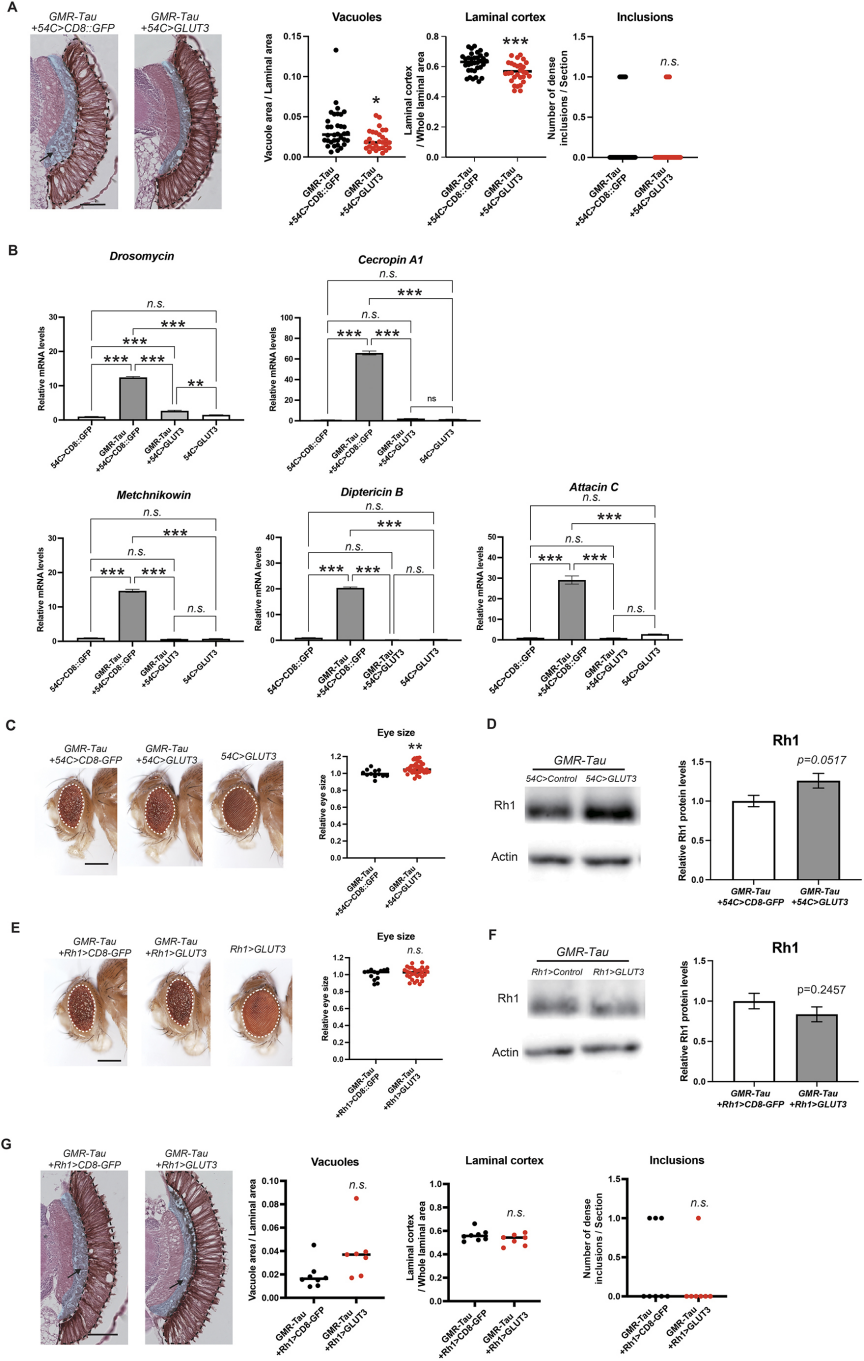
**Enhanced glucose uptake of pigment glia suppresses tau-induced neurodegeneration.** (A-D) Pigment glia-specific GLUT3 expression by using *54C-GAL4* rescued neurodegeneration and glial phenotypes in *GMR-Tau* flies. *GMR-Tau*, which expresses human Tau directly under the control of the GMR regulatory sequence, was combined with GLUT3 expression driven by *54C-GAL4*, a pigment glia-specific driver. CD8::GFP was used as a control for GLUT3. (A) Pigment glia-specific GLUT3 expression suppressed photoreceptor degeneration (Vacuoles) and laminal cortex swelling (Laminal cortex). Data are plotted as the mean±s.e., *n*=29-34, *P<0.05, **P<0.01. Statistical significance was assessed with unpaired two-tailed *t*-test. Scale bar: 100 µm. Flies were used at 7 days after eclosion. (B) GLUT3 expression in the pigment glia suppressed expression of AMPs in *GMR-Tau* flies. Expression of AMPs was quantified using qRT-PCR [B, mean±s.e., *n*=3, **P<0.01, ***P<0.001, one-way ANOVA followed by Tukey's honest significant difference (HSD) multiple comparisons test]. (C-D) GLUT3 expression in the pigment cells suppressed the loss of photoreceptor neurons in *GMR-Tau* flies. (C) Sizes of external eyes (mean±s.e., *n*=12-36, *P<0.05. Statistical significance was assessed with unpaired two-tailed *t*-test. Scale bar: 250 µm) and (D) Rh1 protein levels analyzed by western blotting (mean±s.e., *n*=3, *P*=0.0517. Statistical significance was assessed with unpaired two-tailed *t*-test). (E-G) Photoreceptor neuron-specific GLUT3 expression using *Rh1-GAL4* did not suppress photoreceptor degeneration in *GMR-Tau* flies. *GMR-Tau*, which expresses human Tau directly under the control of the GMR regulatory sequence, was combined with GLUT3 expression driven by *Rh1-GAL4*, a photoreceptor neuron-specific driver. CD8::GFP was used as a control for GLUT3. (E) Sizes of external eyes [mean±s.e., *n*=13-30; n.s., not significant (*P*>0.05). Statistical significance was assessed with unpaired two-tailed *t*-test. Scale bar: 250 µm]. (F) Rh1 protein levels analyzed by western blotting (mean±s.e., *n*=3, *P*=0.2457. Statistical significance was assessed with unpaired two-tailed *t*-test). (G) Photoreceptor neuron-specific GLUT3 expression did not suppress photoreceptor degeneration (Vacuoles) and glial swelling (Laminal cortex). Mean±s.e., *n*=7-8, n.s.; *P*>0.05, one-way ANOVA followed by Tukey's HSD multiple comparisons test. Scale bar: 100 µm.

The regulatory sequences of the *Drosophila ninaE* gene used in *GMR-GAL4* drives expression in all cells behind the morphogenetic furrow, including pigment glial cells (Freeman, 1996). Pigment glial cells surround photoreceptor neurons and support their functions by providing nutrition and by taking up toxic materials (Liu et al., 2017). Expression of *GMR-GAL4* and *54C-GAL4* in pigment glial cells was confirmed by using *UAS-mCh::NLS* (Fig. S4). Although Tau expression in pigment cells does not cause a rough-eye phenotype (Fig. S5), GLUT3 expression in pigment cells might mediate the protective effects against photoreceptor degeneration. To dissect the effects of GLUT3 expression in glial cells during Tau-induced retinal degeneration, we used *GMR-Tau (P301L)* − which expresses a human Tau variant carrying the pathological Pro 301 to Leu mutation (P301L), directly under the control of the GMR regulatory sequence (Jackson et al., 2002) − in combination with the *GAL4/UAS* system to express *GLUT3* under the *54C-GAL4*, a pigment glia-specific driver (Nagaraj and Banerjee, 2007). We found that laminal cortex swelling (Fig. 5A) and expression of AMPs (Fig. 5B) was suppressed by expression of *GLUT3* in pigment glia. Intriguingly, pigment cell expression of *GLUT3* in the Tau-expressing retina also suppressed the degeneration of photoreceptors (Fig. 5A,C). Vacuole formation was suppressed, eye size was significantly increased and protein levels of Rh1 were restored upon *GLUT3* expression in pigment glia cells (Fig. 5A,C,D). In contrast, expression of *GLUT3* in photoreceptor neurons by *Rh1-GAL4* did not mitigate photoreceptor degeneration (Fig. 5E-G), indicating that the protective effects of *GLUT3* coexpression against Tau toxicity observed with *GMR-GAL4* (Fig. 3) was mediated by pigment glia. These results suggest that enhancement of glucose uptake in pigment glia mitigates inflammatory responses and Tau-induced degeneration of photoreceptors.

## DISCUSSION

Enhancing glucose uptake by neuronal overexpression of GLUTs has been reported to ameliorate neurodegeneration in several neurodegenerative conditions, such as cell death caused by expression of huntingtin, β-amyloid or TDP-43 in *Drosophila* (Besson et al., 2015; Manzo et al., 2019; Niccoli et al., 2016; Cabirol-Pol et al., 2018), as well as in age-related neuronal dysfunction (Oka et al., 2021). However, the beneficial effect of enhanced glucose uptake in glial cells has not been examined. In this current study, we characterized glial phenotypes in the retina of flies that express human Tau and examined the effects enhanced glucose uptake has within their retinal pigment glia cells. We found that Tau expression causes phenotypes mediated by glia, such as inclusion formation, swelling of the laminal cortex and expression of AMPs. We also found that the expression of glucose transporters in pigment glia suppresses many of these phenotypes and mitigates photoreceptor degeneration downstream of Tau. Our results suggest that glucose metabolism in glial cells contributes to neuroinflammation*.* The role of glucose metabolism in glia cells to metabolically support neurons has been well established (Morrison et al., 2013; Pellerin and Magistretti, 1994; Chatterjee and Perrimon, 2021). In this study, we revealed an underappreciated role of glucose within pigment glia to regulate their inflammatory responses.

Our study also found that pigment glial cells can function as immune cells in the fly retina, as they are activated in response to Tau-induced lesions. Pigment glia ensheathe each individual ommatidium, the minimum unit of the compound eye, to provide an optical insulation that prevents extraneous light rays from inappropriately activating photoreceptors (Tomlinson, 2012). Pigment glia also accumulates lipid droplets in response to elevated oxidative stress in photoreceptor neurons (Liu et al., 2015), and lipid accumulation has been linked to microglial activation in mammalian microglia (Jung and Mook-Jung, 2020; Marschallinger et al., 2020). Our finding is consistent with the results from a comprehensive single-cell RNA sequence that expression of AMPs overlaps with the pigment glia marker ‘*santa-maria*’ (Li et al., 2022; Yeung et al., 2022). Further characterization of pigment glia to identify the equivalent mammalian glial cell types will make the *Drosophila* retina an *in vivo* platform to study neuroinflammation and neuron-glia interaction.

We found that Tau expression driven by *GMR-GAL4* causes swelling of the laminal cortex, and degeneration of surface and cortex glia, in which *GMR-GAL4* had not been expressed. These non-cell autonomous effects might be mediated by AMPs, since GLUT expression in the pigment glia suppressed expression of AMPs and mitigated the laminal cortex swelling. Although the primary function of AMPs is to kill bacteria (Ganesan et al., 2011), recent reports suggest that AMPs affects diverse physiological processes, such as sleep and memory formation (Toda et al., 2019; Barajas-Azpeleta et al., 2018), implying that AMPs can work as intercellular signaling molecules. In a fly model of traumatic brain injury, downregulation of the NF-κB-signaling pathway in response to mutation of *Relish* or loss of Mtk protects against detrimental effects (Swanson et al., 2020). Secreted AMPs from pigment cells in Tau-expressing retina may damage glial cells in the laminal cortex. Another possible mechanism underlying this non-cell autonomous toxicity may be intercellular lipid transfer. During oxidative stress, neurons release lipids that are internalized by glia, thereby causing them to swell (Liu et al., 2015, 2017). Pigment cells primarily accept lipid droplets from photoreceptor neurons (Liu et al., 2015) but − under neurodegenerative conditions − oxidative stress and lipid shuttling from neurons might exceed the capacity of the pigment cells, and overflowed lipids may harm distal glial cells. Enhanced glucose uptake of pigment cells might enhance their ability to degrade lipid droplets from neurons and reduce the overflow of lipid droplets that affect other glia.

Enhanced glucose uptake in pigment cells suppressed expression of AMPs dramatically; however, photoreceptor degeneration was only mildly rescued. Tau affects many cellular components and processes, such as the cytoskeleton, mitochondria, chromatin structures, lipids and stress signaling that, independently, can contribute to neurodegeneration (DuBoff et al., 2012; Frost et al., 2014; Fulga et al., 2007; Goodman et al., 2024; Papanikolopoulou et al., 2019; Voelzmann et al., 2016). Our results suggest that secretion of AMPs from pigment glia in the Tau-expressing retina is one of the events that, collectively, cause degeneration when induced in parallel within multiple cell types.

Changes in glial activity under disease conditions are often associated with alterations in the metabolic signatures, including glucose metabolism pathways (Edison, 2020; Jha and Morrison, 2018; Wang et al., 2019). Multiple signaling pathways that sense metabolic status are conserved across species and are also known to regulate inflammatory responses (Saito et al., 2019). For example, liver kinase B1 (LKB1, officially known as STK11) is a key regulator of metabolism and activates members of the AMP-activated protein kinase (AMPK) and AMPK-related kinase (ARK) families (Compton et al., 2023), which have been reported to promote anti-inflammatory responses (Compton et al., 2023) by negatively regulating nuclear factor κB (NF-κB) signaling in microglia (Ramamurthy and Ronnett, 2006). Another critical energy regulator, Sirtuin 1 (SIRT1), can suppress NF-κB signaling (Zhang et al., 2020). Many regulatory mechanisms of NF-κB signaling are conserved among species (Buchon et al., 2014), and these pathways can also regulate inflammatory responses in *Drosophila* (Hetru and Hoffmann, 2009). Multiple AMPs downstream of NF-κB signaling are activated upon Tau expression and suppressed upon GLUT3 coexpression. Further studies using metabolic and transcriptomic approaches will facilitate the mechanistic understanding of neuroinflammation.

## MATERIALS AND METHODS

### Fly stocks and husbandry

The following fly stocks were obtained from the Bloomington *Drosophila* Stock Center (BDRC): *UAS-mCherry.NLS* (39434), *Repo-GAL4* (7415), *UAS-mCD8::*ChRFP (27391), *UAS-Luciferase* (35788), *UAS-Luciferase RNAi* (31603), *GMR-Tau* P301L (51377), *UAS-CD8::GFP* (5137), *GMR-GAL4* (9146), *Rh1-GAL4* (8691), and 54C-*GAL4* (27328). *UAS-Glut1^d05758^*, which is a P{XP} insertion containing UAS elements upstream of *Glut1*, was obtained from Harvard Medical School (http://flybase.org/reports/FBti0055936.htm, Exelixis *Drosophila* Collection) (Thibault et al., 2004). *UAS-drpr RNAi* (HMJ30231) and *UAS-NimC4 RNAi* (HMJ23355) were obtained from the Japanese National Institute of Genetics (NIG-FLY). *w^1118^* and *UAS-GLUT3* were a gift from Marie Thérèse Besson (Aix-Marseille Université, Marseille, France) (Besson et al., 2015). *UAS-Tau* (wildtype 0N4R) was a gift from Mel. B. Feany (Brigham and Women's Hospital and Harvard Medical School, Boston, USA) (Wittmann et al., 2001). Flies were reared in a standard medium containing 10% glucose, 0.7% agar, 9% cornmeal, 4% brewer's yeast, 0.3% propionic acid, and 0.1% N-butyl p-hydroxybenzoate [w/v]. Flies were maintained at 25°C under light-dark cycles of 12:12 h. Experiments involving transgenic *Drosophila* were approved by the Tokyo Metropolitan University research ethical committee (#G5-15). Fly genotypes for each experiment are listed in Tables S1 and S2.

### Measurement of eye size

Images of fly eyes were captured with an Olympus digital microscope DSX110 or the Leica MZ16 stereomicroscope, and the area of the eye surface was measured using Fiji (NIH). Eyes from more than 11 flies were analyzed for each genotype.

### Immunostaining

Flies were collected at 3-5 days old, and brains were dissected in PBS, fixed in 4% PFA/PBS (Thermo Scientific) for 30 min at room temperature (RT). Brains were washed with PBST for 10 min three times. Normal donkey serum at 4% (abcam) was used as a blocking buffer for 30 min at RT. Brains were stained with antibody against *Drosophila* elav (1:1000, DSHB cat# Elav-9F8A9, RRID:AB_528217), Repo (1:50, DSHB cat# 8D12, RRID:AB_528448) or ubiquitin (FK2, 1:1000, StressMarq Biosciences cat# SMC-214D-DY405, RRID:AB_2820835) and visualized with goat anti-rat antibody conjugated to Alexa Fluor 488 (1:1000, Thermo Fisher Scientific cat# A-11006, RRID:AB_2534074) or goat anti-mouse antibody conjugated to Alexa Fluor 488 (1:1000, (Thermo Fisher Scientific cat# A-11001, RRID:AB_2534069). Nuclei were stained with DAPI (1:1000, abcam). Filamentous actin was stained with Alexa Flour 488 Phalloidin (1:1000, Invitrogen). Brains were mounted in VectaShield (Vector Laboratories) and imaged using Nikon spinning-disc confocal microscope.

### Western blotting

Flies were frozen in liquid nitrogen, and fifteen fly heads per genotype were homogenized in SDS-Tris-Glycine sample buffer. The same amount of the lysate was loaded to each lane of multiple 10% Tris-Glycine gels and transferred to PVDF membrane. Membranes were blocked with 5% skimmed milk in tris-buffered saline with 0.1% Tween 20 (TBST), blotted using the antibodies described below, incubated with appropriate secondary antibodies and detected using Immobilon Western Chemiluminescent HRP Substrate (Merck Millipore). Membranes were probed with anti-actin antibody (Sigma) as the loading control. Antibodies against Tau pS202 (CP13) and phosphorylated-Ser396/404-Tau (PHF1) were a gift from Peter Davis (Albert Einstein College of Medicine, New York, USA). Antibodies against Tau (T46, Thermo Fisher Scientific cat# 13-6400, RRID:AB_2533025), posphorylated-Ser262-Tau (pSer262, abcam, cat# ab92627, RRID:AB_10563129) rhodopsin 1 (4c5, DSHB cat# 4c5, RRID:AB_528451) and actin (Sigma-Aldrich cat# A2066, RRID:AB_476693) were purchased. The chemiluminescent signals were detected by using Fusion FX software (Vilber) and intensity was quantified with Fiji (NIH). Western blots were repeated at least three times with different animals, and representative blots are shown. Flies used for western blotting were 5-7 days old after eclosion.

### Histological analysis

Heads of flies aged 3-5 days after eclosion were fixed in Bouin's fixative for 48 h at RT and incubated for 24 h in a leaching buffer (50 mM Tris/150 mM NaCl). Samples were dehydrated in a series of ethanol baths (70%, 80%, 95% and 100%) and xylene at RT, and embedded in paraffin. Serial sections (6 μm thick) through the entire heads were stained with hematoxylin and eosin (H&E). Sections were mounted to glass slides, de-paraffined in xylene and a series of ethanol baths (100%, 95%, 80%) for 3 min each. Slides were stained with 100% hematoxylin for 2 min and washed in tap water for 5 min. They were then stained with 100% eosin for 30-45 s, washed with 95% ethanol and 100% ethanol for 6 min each, incubated with xylene for 10 min, dried and mounted with a coverslip. Each section was examined using bright-field microscopy. Images of the sections that include the lamina were taken using a BZ-X700 (Keyence) or Mica (Leica) microscope. Retinal degeneration or the vacuole area was analyzed using Fiji (NIH). The area of the laminal cortex was measured and normalized to the whole laminal area to calculate the laminal cortex area.

### Electron microscopy

Decapitated heads were cut in half vertically, then incubated in primary fixative solution (2.5% glutaraldehyde and 2% paraformaldehyde in 0.1 M sodium cacodylate buffer) at RT for 2 h. After washing heads with 3% sucrose in 0.1 M sodium cacodylate buffer, fly heads were post-fixed for 1 h in secondary fixation (1% osmium tetroxide in 0.1 M sodium cacodylate buffer) on ice. After washing with H_2_O, heads were dehydrated with ethanol and infiltrated with propylene oxide and Epon mixture (TAAB and Nissin EM) for 3 h. After infiltration, specimens were embedded with an Epon mixture at 70°C for 2∼3 days. Some longitudinal sections cut out by glass knives were stained with toluidine blue to check the position. Thin sections (70 nm) of retinal sections were collected on copper grids. The sections were stained with 2% uranyl acetate in 70% ethanol and Reynolds' lead citrate solution. Electron micrographs were obtained with a VELETA CCD Camera (Olympus Soft Imaging Solutions GMBH) mounted on a JEM-1010 electron microscope (Jeol Ltd.).

### RNA extraction and quantitative real-time PCR analysis

Heads from more than 25 flies were mechanically isolated, and total RNA was extracted using ISOGEN (NipponGene), followed by reverse transcription using the PrimeScript RT reagent kit (Takara). The resulting cDNA was used as a template for PCR with THUNDERBIRD SYBR qPCR mix (TOYOBO) on a Thermal Cycler Dice real-time system TP800 (Takara). Expression of genes of interest was standardized relative to *rp49* or *actin*. Relative expression values were determined by the ΔΔCT method (Livak and Schmittgen, 2001). Experiments were repeated three times, and a representative result was shown. Primers were designed using DRSC FlyPrimerBank (Harvard Medical School). Primer sequences are: *Attacin C* for 5′-CTGCACTGGACTACTCCCACATCA-3′ and *Attacin C* rev 5′-CGATCCTGCGACTGCCAAAGATTG-3′, *Cecropin A1* for 5′-CATTGGACAATCGGAAGCTGGGTG-3′ and *Cecropin A1* rev 5′-TAATCATCGTGGTCAACCTCGGGC-3′, *Diptericin B* for 5′-AGGATTCGATCTGAGCCTCAACGG-3′ and *Diptericin B* rev 5′-TGAAGGTATACACTCCACCGGCTC-3′, *Drosomycin* for 5′-AGTACTTGTTCGCCCTCTTCGCTG-3′ and *Drosomycin* rev 5′-CCTTGTATCTTCCGGACAGGCAGT-3′, *Metchnikowin* for 5′-CATCAATCAATTCCCGCCACCGAG-3′ and *Metchnikowin* rev 5′-AAATGGGTCCCTGGTGACGATGAG-3′, *drpr* for 5′-GCAGATGCCTGAATAACTCCTC-3′ and *drpr* rev 5′-TCCTTGCATTCCATGCCGTAG-3′, *NimC4* for 5′-GAACGAGACGATACGAGCCAC-3′ and *NimC4* rev 5′-GGTGACTTGTTCCTCCTCTGA-3′, *Glut1* for 5′-TTACCGCGGAGCTCTTCTCC-3′ and *Glut1* rev 5′-GCCATCCAGTTGACCAGCAC-3′, *rp49* for 5′-GCTAAGCTGTCGCACAAATG-3′ and *rp49* rev 5′-GTTCGATCCGTAACCGATGT-3′, *actin 5C* for 5′-TGCACCGCAAGTGCTTCTAA-3′ and *actin 5C* rev 5′-TGCTGCACTCCAAACTTCCA-3′.

## Supplementary Material

10.1242/dmm.052057_sup1 Supplementary information
