# Supplementary material for: Glucose uptake in pigment glia suppresses Tau-induced inflammation and photoreceptor degeneration
Source: Dis Model Mech. 2025 Apr 29;18(4):dmm052057. doi: 10.1242/dmm.052057 (PMC12067088; doi:10.1242/dmm.052057)
Supplement: Supplementary information [file dmm-18-052057-s1.pdf]

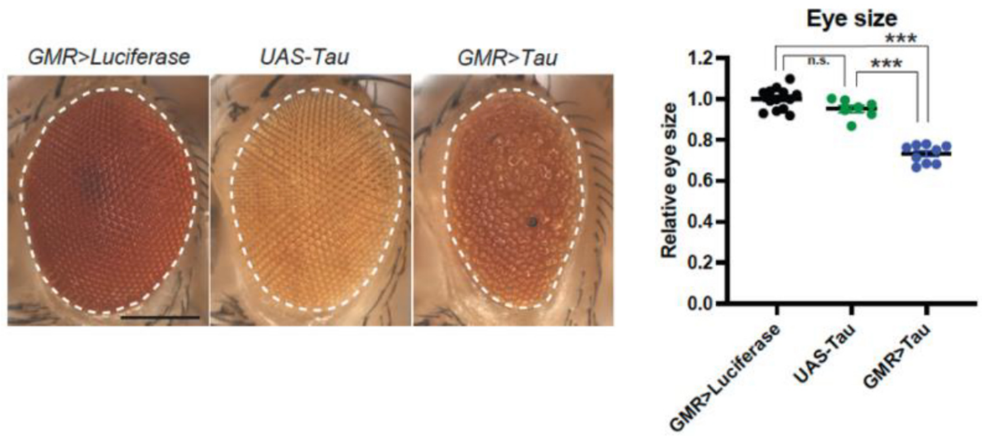

Fig. S1. ***UAS-Tau*** **alone does not reduce eye size**. The area of the eyes was similar between *GMR>Luciferase* and UAS-tau, and that of *GMR> Tau* was significantly less. Scale bar: 250 µm. Mean±SE, n=7-13, ***; p<0.001, n.s.; p>0.05, One-way ANOVA followed by ~0's HSD Tukey's comparisons test.

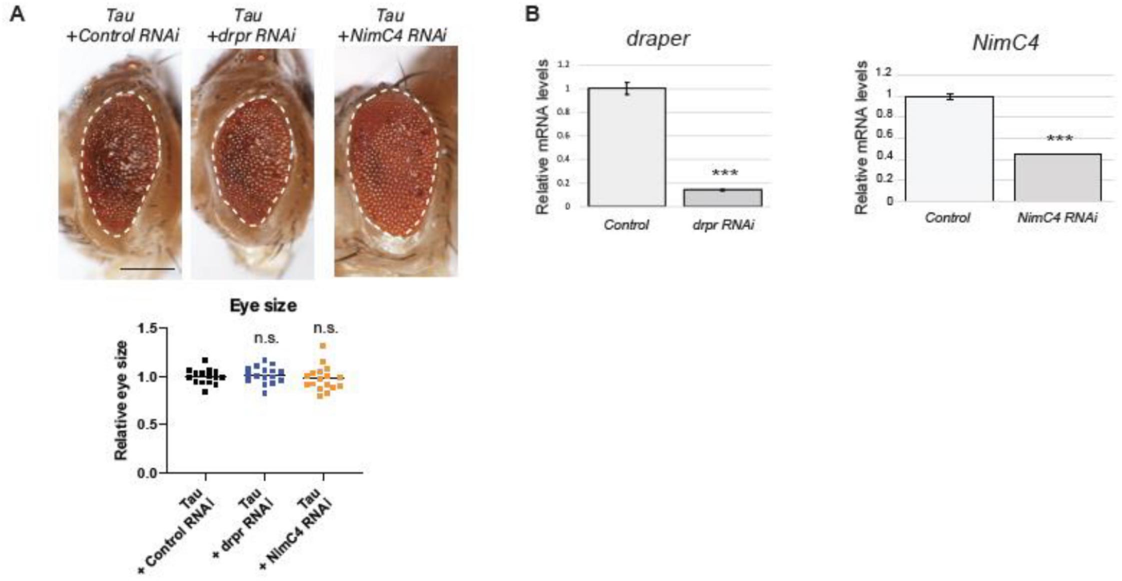

Fig. S2. Knockdown of *drpr* or *NimC4* in tau-expressing retina does not affect eye size. (A) Fly heads expressing *UAS-drpr* RNAi or *UAS-NimC4* RNAi driven by *GMR-GAL4* were subjected to qRT-PCR. Mean±SE, n=3, ***; p<0.001, Statistical significance was assessed with unpaired two-tailed t-test. (B) The eyes of flies co-expressing Tau and *drpr* RNAi or *NimC4* RNAi. *mCherry* RNAi was used as a control. Scale bar: 250 µm. Mean±SE, n=15-17, n.s.; p>0.05, One-way ANOVA followed by Tukey's HSD multiple comparisons test.

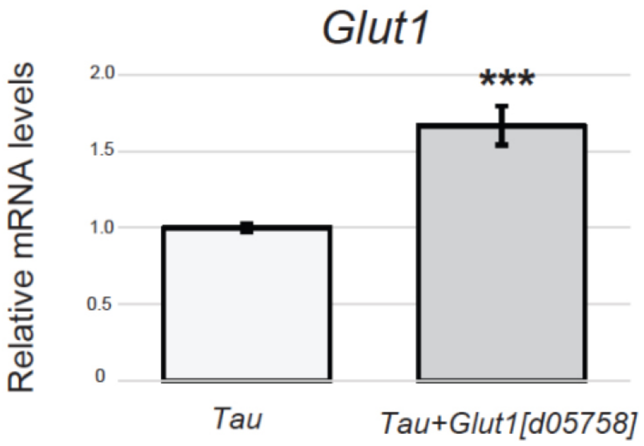

Fig. S3. Overexpression of Glut1 with UAS-Glut1^{d05758} expression driven by *GMR-GAL4*. Heads of the flies with *UAS-Glut1^{d05758}* expression driven by *GMR-GAL4* were subjected to qRT-PCR. Mean±SE, n=3, ***; p<0.001, Statistical significance was assessed with unpaired two-tailed t-test.

Disease Models & Mechanisms • Supplementary information

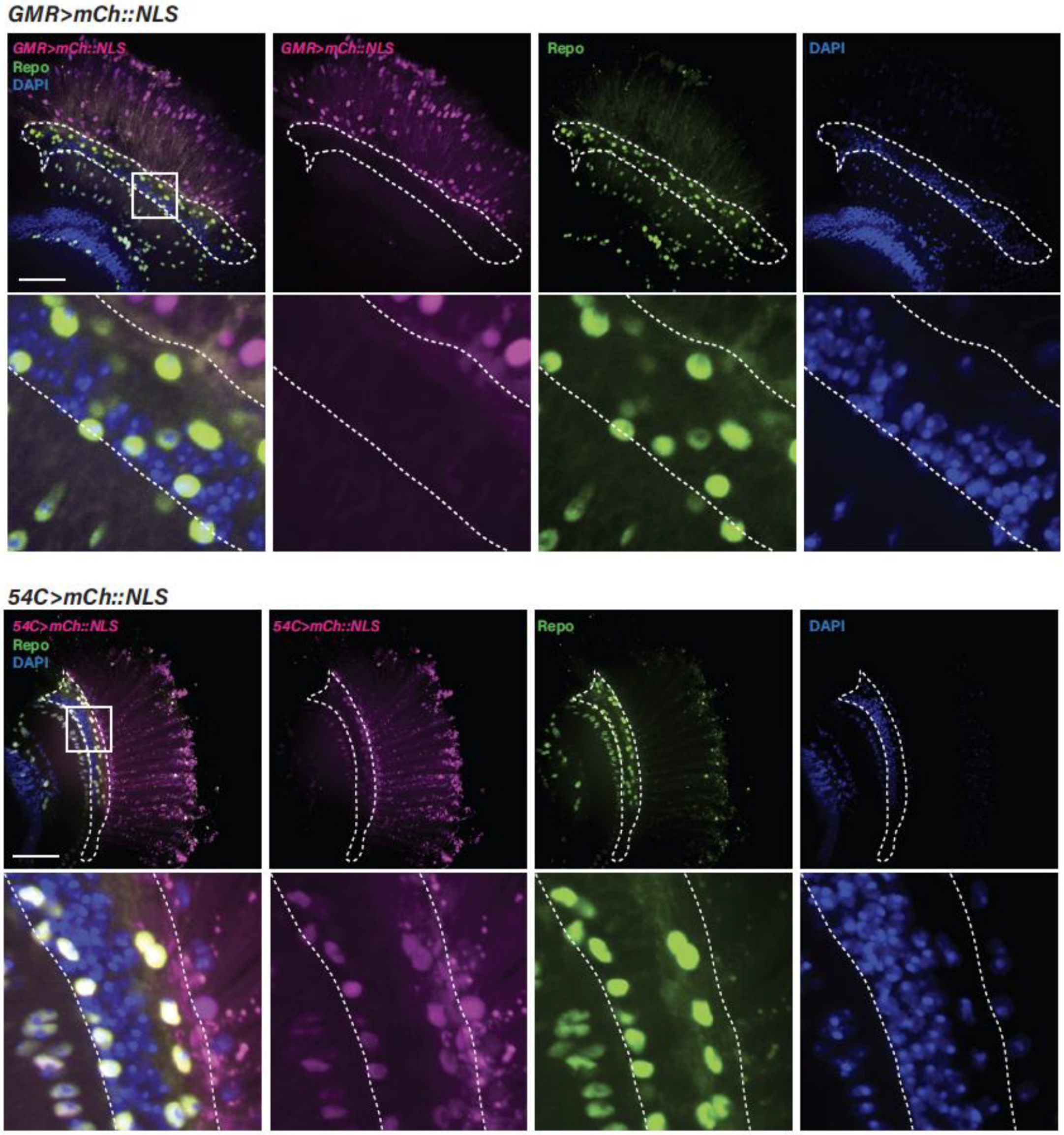

Fig. S4. Expression patterns of *GMR-GAL4* and *54C-GAL4*. *GMR-GAL4*- or *54C-GAL4*-expressing cells are labeled with expression of *UAS-mCh::NLS* (magenta). Glial cells are immunostained by anti-repo antibody (green). White dashed lines indicate the laminal cortex. Scale bars: 50 µm.

Disease Models & Mechanisms • Supplementary information

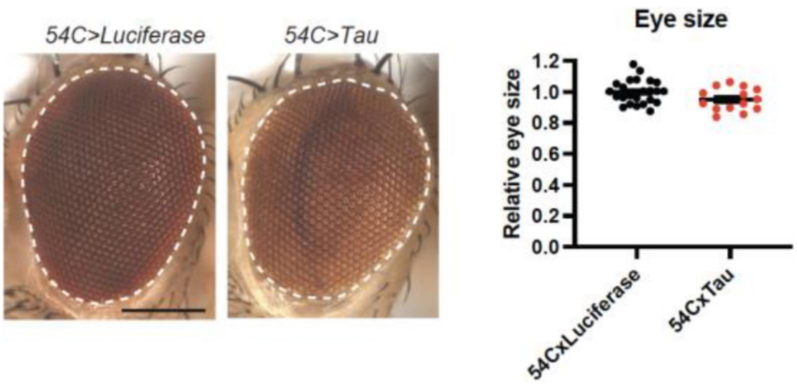

Fig. S5. The eyes with Tau expression driven by *54C-GAL4*. Tau expression was driven by 54C-GAL4. Luciferase was used as a control (*54C>Luciferase*). No significant difference was found in the area of the eye. Scale bar: 250 µm. Mean±SE, n=14-24, n.s.; p>0.05, Statistical significance was assessed with unpaired two-tailed t-test.

Disease Models & Mechanisms • Supplementary information

Table S1. The genotypes of flies used in each experiment related to Figures 1-5

Figure	Name	*Genotype*
Figure 1A-C, 1E-F and G, left	Control	*w^{1118}; GMR-GAL4/+*
	Tau	*w^{1118}; GMR-GAL4/+; UAS-tau/+*
Figure 1D	Control	*w^{1118};; Repo-GAL4/UAS-mCh::NLS*
	Tau	*GMR-Tau/+;; Repo-GAL4/UAS-mCh::NLS*
Figure G, right	Tau	*GMR-Tau/+*
Figure 2A	Tau+Control RNAi	*w^{1118}; GMR-GAL4/+; UAS-tau/UAS-Luciferase RNAi*
	Tau+drpr RNAi	*w^{1118}; GMR-GAL4/UAS-drpr RNAi; UAS-tau/+*
	Tau+NimC4 RNAi	*w^{1118}; GMR-GAL4/UAS-NimC4 RNAi; UAS-tau/+*
Figure 2B	Control	*w^{1118}; GMR-GAL4/+*
	Tau	*w^{1118}; GMR-GAL4/+; UAS-tau/+*
Figure 3A	Control	*w^{1118}; GMR-GAL4/+*
	Tau	*w^{1118}; GMR-GAL4/+; UAS-tau/+*
	Tau+GLUT3	*w^{1118}; GMR-GAL4/UAS-GLUT3; UAS-tau/+*
	Tau+CD8::RFP	*w^{1118}; GMR-GAL4/UAS-mCD8::ChRFP; UAS-tau/+*
	GLUT3	*w^{1118}; GMR-GAL4/UAS-GLUT3*
Figure 3B-F	Control	*w^{1118}; GMR-GAL4/+*
	Tau	*w^{1118}; GMR-GAL4/+; UAS-tau/+*
	Tau+GLUT3	*w^{1118}; GMR-GAL4/UAS-GLUT3; UAS-tau/+*
Figure 3G-I	Control	*w^{1118}; GMR-GAL4/UAS-mCD8::ChRFP*
	Tau	*w^{1118}; GMR-GAL4/ UAS-mCD8::ChRFP; UAS-tau/+*
	Tau+Glut1^{d05758}	*w^{1118}; GMR-GAL4/+; UAS-tau/ UAS- Glut1^{d05758}*
	Glut1^{d05758}	*w^{1118}; GMR-GAL4/+; UAS- Glut1^{d05758}/+*
Figure 4	Tau	*w^{1118}; GMR-GAL4/+; UAS-tau/+*
	Tau+GLUT3	*w^{1118}; GMR-GAL4/UAS-GLUT3; UAS-tau/+*
Figure 5A-D	54C>CD8::GFP	*w^{1118}; 54C-GAL4/ UAS-mCD8::GFP*
	GMR-Tau +54C>CD8::GFP	*GMR-Tau/+; 54C-GAL4/UAS-mCD8::GFP*
	GMR-Tau	*GMR-Tau/+; 54C-GAL4/UAS-GLUT3*

	+54C>GLUT3	
	54C>GLUT3	*w^{1118}; 54C-GAL4/UAS-GLUT3*
Figure 5E-G	Rh1>CD8::GFP	*w^{1118}; UAS-mCD8::GFP /+; Rh1-GAL4/+*
	GMR-Tau +Rh1>CD8::GFP	*GMR-Tau/+; UAS-mCD8::GFP/+; Rh1-GAL4/+*
	GMR-Tau +Rh1>GLUT3	*GMR-Tau/+; UAS-GLUT3/+; Rh1-GAL4/+*
	Rh1>GLUT3	*w^{1118}; UAS-GLUT3/+; Rh1-GAL4/+*

Table S2. The genotypes of flies used in each experiment related to Supplemental Figures 1-5

Figure	Name	*Genotype*
Supplemental Figure 1	GMR>Luciferase	*w^{1118}; GMR-GAL4/+; UAS-Luciferase/+*
	UAS-Tau	*w^{1118};; UAS-Tau/+*
	GMR>Tau	*w^{1118}; GMR-GAL4/+; UAS-Tau/+*
Supplemental Figure 2	Control	*w^{1118}; GMR-GAL4/+; UAS-Luciferase RNAi/+*
	drpr RNAi	*w^{1118}; GMR-GAL4/UAS-drpr RNAi*
	NimC4 RNAi	*w^{1118}; GMR-GAL4/UAS-NimC4 RNAi*
Supplemental Figure 3	Tau	*Elav-GAL4/+; UAS-mCD8::GFP/+; UAS-tau/+*
	Tau+Glut1	*Elav-GAL4/+;; UAS-tau/ UAS- $Glut1^{d05758}$*
Supplemental Figure 4	GMR>mCh::NLS	*w^{1118}; GMR-GAL4/+; UAS-mCh::NLS/+*
	54C>mCh::NLS	*w^{1118}; GMR-GAL4/54C-GAL4*
Supplemental Figure 5	54C>Luciferase	*w^{1118}; 54C-GAL4/+; UAS-Luciferase/+*
	54C>Tau	*w^{1118}; 54C-GAL4/+; UAS-Tau/+*

Disease Models & Mechanisms • Supplementary information
